# Prospective case‐control study of enterovirus detection differences in children’s cerebrospinal fluid between multiplex PCR and real‐time RT‐PCR assay

**DOI:** 10.1002/jcla.23606

**Published:** 2020-11-04

**Authors:** Dianping You, Fang Chen, Jingjie Li, Xianping Zeng, Weijian Wang, Yinghui Guo, Fan Yang, Suzhen Sun, Le Wang

**Affiliations:** ^1^ Institute of Pediatric Research Children’s Hospital of Hebei Province Shijiazhuang China; ^2^ Department of Neurology Children’s Hospital of Hebei Province Shijiazhuang China; ^3^ Ningbo Health Gene Tech Co.Ltd Ningbo China

**Keywords:** children, enterovirus, multiplex PCR, real‐time PCR

## Abstract

**Background:**

Viral encephalitis is common in childhood. It is an acute brain parenchymal inflammation caused by a variety of viral infection, and enterovirus accounts for the majority. Due to atypical clinical manifestations, pathogenic testing is important for assisting clinical diagnosis. The purpose of this study was to evaluate the performance of the multiplex PCR assay compared with quantitative real‐time PCR for enterovirus detection.

**Methods:**

A prospective case‐control study was performed involving 103 pediatric patients suspected for viral encephalitis and cerebrospinal fluid (CSF) samples were collected and tested for 9 pathogens using multiplex PCR assay during April to November in 2018. In parallel, an aliquot of samples was tested for enterovirus infection by real‐time PCR assay.

**Results:**

There were 85.4% children were confirmed as viral encephalitis on discharge, the remaining ones were diagnosed as other CNS diseases, such as epilepsy. The specificity of the two methods was the same as that of the clinical diagnosis, but the sensitivity and consistency with clinical diagnosis of multiplex PCR were both higher than the real‐time PCR. Besides of enterovirus, multiplex PCR could also detect coinfection of enterovirus with Epstein‐Barr virus and mumps virus.

**Conclusion:**

Results of multiplex PCR method are more consistent with the clinical diagnosis and are superior to real‐time PCR for detecting enterovirus in CSF.

## Graphical abstract

A comparison study between pathogen detection of viral encephalitis was conducted in a case‐control manner. The specificity of the two methods was the same as that of the clinical diagnosis, but the consensus value and sensitivity of the multiplex PCR were both higher than the real‐time PCR.

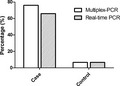

**Abbreviations**
CMV: cytomegalovirus
EBV: Epstein‐Barr virus
EV: Enterovirus
HHV6: human herpesvirus type 6
HSV‐1: herpes simplex virus type 1
HSV‐2: herpes simplex virus type 2
MuV: mumps virus
VZV: varicella‐zoster virus

## 1 BACKGROUND

Enterovirus (EV)‐induced viral meningitis in infants and young children can cause severe morbidity and mortality^1^ and is a common cause of hospital admission, especially during the summer.^2^ Proper immediate diagnosis and initiation of specific, evidence‐based antiviral therapy is essential for survival and reduces the likelihood of permanent brain damage. However, it is difficult or time‐consuming to differentiate viral meningitis from other infections clinically.^3^, ^4^

It is well known that enterovirus infection accounts for a proportion of acute encephalitis in children, but it is difficult to distinguish it from other viruses based only on clinical manifestation or surrogate markers in CSF (eg, white cell count and pleocytosis).^5^ Emerging multiplex PCR is very useful to help overcome some of the challenges.^3^, ^6^ At the same time, several other groups have also designed single‐targeted real‐time PCR for early detection of DNA or RNA of common viruses in CSF.^7^ However, it has been reported that the sensitivity of multiplex PCR is inferior than that of single‐targeted real‐time PCR.^8^ In addition, the role of molecular diagnostic testing in clinical applications remains unclear, as early studies focused solely on patients with confirmed infection, while the control group was not included.^9^

Therefore, in the present study, our aim was to compare the clinical diagnostic coincidence rates of multiplex PCR and real‐time PCR to test for enterovirus infection in children hospitalized for acute viral encephalitis and other central nervous system diseases.

## 2 MATERIALS AND METHODS

### 2.1 Study samples

The research protocol, collection, and use of clinical data were approved by the Research Ethics Board, Children's Hospital of Hebei Province. From April to November 2018, a total of 103 CSF specimens were collected from hospitalized patients diagnosed with suspected viral encephalitis within 48 hours of admission. A patient is defined as an viral encephalitis suspected case if (1) he/she had symptoms and signs of CNS infection, including acute onset, plus at least one symptom of fever, headache, or vomiting plus either meningeal signs or changes in mental status; (2) cerebral parenchymal abnormalities in neuroimaging of encephalitis or EEG abnormalities consistent with encephalitis; (3) no evidence of bacterial meningitis by microscopy and CSF culture; (4) clear appearance of cerebrospinal fluid, normal, or mildly moderate white blood cell count, glucose, chloride, and protein. Patients with metabolic, toxic, or neurological degenerative diseases will be excluded.

Obtained by lumbar puncture, CSF samples were collected and used for routine CSF biochemical tests and culture, the remaining samples were stored at −80°C for molecular analysis.

After treatment and observation, patients who were finally diagnosed with other central nervous diseases were enrolled into the control group to evaluate the molecular diagnostic assay.

### 2.2 DNA/RNA extraction

A total of 200 µL CSF samples were used to extracted and purified nucleic acid by extraction kit (HGT, Ningbo, China) on an automated extraction workstation Smart LabAssist‐16/32 (TANBead, Taiwan, China). The extracts were immediately used as template for PCR amplification or stored below −20°C.

### 2.3 Detection of pathogens by multiplex PCR and qPCR

The one‐step RT‐PCR was fulfilled with the ABI Verity 96 Thermal Cycler. The PCR products were added to a 96‐well plate, prepared for capillary electrophoresis (CE), and fragment analysis by applying the 3500 Genetic Analyzer (ABI, USA), according to the manufacturer's protocol. The multiplex PCR panel included *Mycoplasma pneumoniae* and 8 viruses: enterovirus (EV), varicella‐zoster virus (VZV), mumps virus (MuV), cytomegalovirus (CMV), herpes simplex virus type 1 (HSV‐1), herpes simplex virus type 2 (HSV‐2), Epstein‐Barr virus (EBV), and human herpesvirus type 6 (HHV6).

The RT‐PCR was used to detect EV in CSF samples. The ABI 7500 real‐time PCR thermal cycler (Thermo Fisher, USA) and Real‐time TaqMan PCR reagents (Da'an gene Tech, China) were used to amplify the five targets separately according to the manufacturer's instructions.

### 2.4 Statistical analysis

Chi‐square test was used on the SPSS 13.0.1 statistics package (SPSS Inc, Chicago, USA). Agreement of the results between molecular assay and discharge diagnosis was assessed using Kappa statistics (κ value 0.21‐0.4 fair, 0.41‐0.6 moderate, 0.61‐0.8 substantial, and 0.81‐1 almost perfect).^10^ *P* < .05 was considered statistically significant.

## 3 RESULTS

### 3.1 Study population

A total of 103 CSF specimens enrolled in this study (Table 1), including 88 CSF samples collected from infants and children (51 males, 37 females) who had a discharge diagnosis of viral encephalitis. 87.5% (77/88) patients were observed to have upper respiratory infection symptoms. A total of 15 CSF specimens were from inpatient children (9 males, 6 females) who were eventually diagnosis with other CNS diseases, such as epilepsy (Table 2).

**TABLE 1:** Demographics of pediatric patients with suspected viral encephalitis

Male	Female	Total	Interquartile range of age (years)
60	43	103	5.9 (4‐8)

**TABLE 2:** The diagnosis at discharge

Diagnosis	Number	Percentage (%)
Viral encephalitis	88	85.4
With upper respiratory tract infection	77	74.8
Other CNS diseases	15	14.6
Epilepsy	3	2.9
Febrile convulsion	2	1.9
Purulent meningitis	2	1.9
Neurosis	2	1.9
Autoimmune encephalitis	2	1.9
Intracranial hypertension	1	1.0
Systemic inflammatory response syndrome	1	1.0
Central nervous system demyelination	1	1.0
Acute tonsillitis	1	1.0

### 3.2 Clinical concordance with multiplex PCR and real‐time PCR

A moderate agreement (κ value = 0.447) was observed between the discharge diagnosis and multiplex PCR results, but a fair agreement (κ value = 0.329) was observed between the discharge diagnosis and real‐time PCR. In the CSF from one case, both multiple PCR and RT‐PCR tests showed positive EV, but the discharge diagnosis was epilepsy. In addition, 21 and 30 cases were diagnosed as viral encephalitis without certain pathogen detection using these two methods, respectively (Table 3). The false‐negative rate shown by multiplex PCR was significantly lower than that of real‐time PCR (*P* = .013).

**TABLE 3:** Clinical agreements with multiplex PCR and real‐time PCR, respectively

		Viral Encephalitis	Control			Viral Encephalitis	Control
multiplex PCR	EV (+)	67	1	qPCR	EV (+)	58	1
	EV (−)	21	14		EV (‐)	30	14
Sensitivity		76.1%		Sensitivity		65.9%	
Specificity		93.3%		Specificity		93.3%	
Kappa value		0.447		Kappa value		0.329	
False‐positive case		1		False‐positive case		1	
False‐negative case		21		False‐negative case		30	

Note

*P* = .013 by McNemar's test.

### 3.3 Co‐detection by multiplex PCR

Besides of EV, multiplex PCR assay also identified other viruses, including 1 EBV and 4 MuV. Seven mixed infections (EV and EBV) were also identified by multiplex PCR (Table 4).

**TABLE 4:** The other viruses detection by the multiplex PCR assay

	Number	Percentage (%)
Single virus detected	66	64.1
EV	61	59.2
EBV	1	1.0
MuV	4	3.9
Double viruses detected	7	6.8
EV & EBV	7	6.8

Abbreviations:

EV, enterovirus; EBV, epstein‐barr virus; MuV, mumps virus.

## 4 DISCUSSION

Nucleic acid amplification techniques (NAATs) such as real‐time PCR and multiplex PCR have been widely used to identify pathogens in infectious central nervous system diseases.^11^, ^12^ These NAATs prevent misdiagnosis in children with normal cellularity, normal protein levels, or without hypoglycorrhachia, whose CSF PCR tested positive for EV.^13^, ^14^ These data highlight the need to perform PCR in CSF of children despite the normal results of the traditional tests. However, Only a few reports have described the performance of NAATs in microbiological testing in pediatric patients suspected of viral encephalitis.^6^, ^15^, ^16^ The clinical application of single‐targeted NAATs is limited due to the insufficient CSF volume and small number of detection channels. On the other hand, methodological studies indicated that multiplex PCR may be less sensitive than the corresponding single‐targeted real‐time PCR due to the imbalance in amplification efficiency between diverse targets.^8^ Therefore, it is necessary to use CSF from children with viral encephalitis to compare the differences between the two methods.

In this study, we compared the detection of EV in 103 CSF specimens from hospitalized children with suspected viral encephalitis in the summer months by real‐time PCR and multiplex PCR, and we found a higher sensitivity of multiplex PCR. In addition, we used discharge diagnosis as a standard, a moderate diagnosis agreement of multiplex PCR, but a fair agreement of real‐time PCR was observed, respectively.

Only a few studies compared the different NAATs assay to test viral yield of CSF samples. Crom et al measured enterovirus (EV) and human parechovirus (HPeV) by GeneXpert and real‐time PCR on 116 CSF samples collected from patients with meningitis symptoms. They found that these two molecular assays were superior to viral culture for detecting EV in CSF, and real‐time PCR performed better than GeneXpert in detecting EV infection.^17^ Wong tested 3 types of viruses, that is, HSV‐1/2 and VZV in 150 children with viral encephalitis using multiplex RT‐PCR kit, and revealed that the multiplex assay showed excellent sensitivity, specificity, and reproducibility when compared to the single‐plex real‐time PCR assay.^18^ Similar to our study, multiplex PCR was more sensitive than real‐time PCR (80.7% vs. 65.9%). Generally, the sensitivity of multiplex PCR is one log lower than real‐time PCR,^19^, ^20^ but the method we used combines multiplex PCR with capillary electrophoresis separation technology, which can indeed achieve higher sensitivity due to the following reasons. (1) Through optimizing the primer sequences, the generation of primer dimers is reduced, and the amplification efficiency of certain targets can be equivalent to single‐plex PCR. (2) Capillary electrophoresis can separate fluorescent primers, primer dimers, and specific amplification products, so that the fluorescent signals of specific products are not interfered by the background signals. In addition to EV, we also detected EBV, MuV, and coinfection by multiplex PCR. Therefore, the combing detection of multiple targets in a single reaction is particularly valuable for adapting to insufficient CSF volumes obtained from some children with multiple microbiological test requests and reducing turn‐around‐times and costs. Based on these findings, multiplex PCR could reasonably replace the single‐targeted PCR as an inpatient procedure for children to avoid missed diagnosis of viral infection.

In our work, some patients were clinically diagnosed with viral encephalitis, but no viral infection was detected by multiplex PCR or real‐time PCR. Similarly, this was also observed in another multiplex PCR assay named Seeplex Meningitis ACE, where 43.6% (34/78) CSF findings were consistent with bacterial or viral infections, but multiplex PCR results were negative.^21^ The reason for this “false‐negative” may be that the encephalitis is caused by a pathogen other than the target in test kit. Alternatively, the concentration of pathogens in CSF may be too low to permit detection. In addition to false‐negative cases, the false‐positive ones were also observed, as one patient was diagnosed with epilepsy and both multiplex PCR and real‐time PCR showed positive enterovirus results. It is well known that CNS infection is the main risk factor for epilepsy.^22^ Approximately 42% of infants with enterovirus infection present with severe seizures.^23^ When status epilepticus is accompanied by encephalitis, the prognosis is worse than etiologies infection,^24^ Therefore, for such patients, it is more necessary to understand the pathogens in CSF.

Furthermore, in our and others’ studies, the presence of mixed pathogen is remarkable. We observed 6.8% (7/103) coinfection as EV and EBV, and Kahraman et al found that 9.1% (3/33) CSF samples were simultaneously positive for 2 pathogens.^25^ Shin et al found a case was positive for *L monocytogenes* and EBV by multiplex PCR, but only positive for *L monocytogenes* by conventional PCR.^21^ These data suggest that multiplex PCR methods may increase the isolation rate of pathogens in central nervous system infections. Further research is needed to investigate the clinical relevance of this coinfection result.

## 5 CONCLUSIONS

EV was the most identified virus causing meningitis in children. It is needed to applicate viral PCR testing in clinical. In this study, we observed a higher sensitivity and a higher consistency of clinical diagnosis of multiplex PCR compared with single‐target real‐time PCR. The results of rapid multiplex PCR testing can be used to guide antimicrobial therapy and may result in reduced antimicrobial exposure in children with viral encephalitis.

## CONFLICT OF INTERESTS

The authors declare that they have no competing interests.

## AUTHORS’ CONTRIBUTIONS

LW and SZS designed the study and take responsibility for the entire process; DPY and FC conducted literature search, data extraction, quality assessment, and draft writing; JJL, YHG, and FY collected and analyzed the data; XPZ and WJW edited the article. All authors have read and approved the final article.

## ETHICS APPROVAL AND CONSENT TO PARTICIPATE

The study was approved by the Children's hospital Hebei Province Ethics Committee (number 2 018 002). The legal guardian(s) or parent(s) of the children provided written informed consent for sample collection and clinical record review.

## Data Availability

The datasets generated and/or analyzed during the current study are available in the (Figshare) repository, (https://figshare.com/articles/ViralEncephaCompare/8312780).
